# Feasibility and Acceptability of Engaging Care Partners of Persons Living With Dementia With Electronic Outreach for Deprescribing

**DOI:** 10.1093/geront/gnaf028

**Published:** 2025-01-28

**Authors:** Katharina Tabea Jungo, Niteesh K Choudhry, Edward R Marcantonio, Gauri Bhatkhande, Katherine L Crum, Nancy Haff, Kaitlin E Hanken, Julie C Lauffenburger

**Affiliations:** Institute of Primary Health Care (BIHAM), University of Bern, Bern, Switzerland; Center for Healthcare Delivery Sciences, Department of Medicine and Division of Pharmacoepidemiology and Pharmacoeconomics, Brigham and Women’s Hospital, Boston, Massachusetts, USA; Harvard Medical School, Boston, Massachusetts, USA; Center for Healthcare Delivery Sciences, Department of Medicine and Division of Pharmacoepidemiology and Pharmacoeconomics, Brigham and Women’s Hospital, Boston, Massachusetts, USA; Harvard Medical School, Boston, Massachusetts, USA; Harvard Medical School, Boston, Massachusetts, USA; Divisions of General Medicine and Gerontology, Department of Medicine, Beth Israel Deaconess Medical Center, Boston, Massachusetts, USA; Center for Healthcare Delivery Sciences, Department of Medicine and Division of Pharmacoepidemiology and Pharmacoeconomics, Brigham and Women’s Hospital, Boston, Massachusetts, USA; Center for Healthcare Delivery Sciences, Department of Medicine and Division of Pharmacoepidemiology and Pharmacoeconomics, Brigham and Women’s Hospital, Boston, Massachusetts, USA; Center for Healthcare Delivery Sciences, Department of Medicine and Division of Pharmacoepidemiology and Pharmacoeconomics, Brigham and Women’s Hospital, Boston, Massachusetts, USA; Harvard Medical School, Boston, Massachusetts, USA; Center for Healthcare Delivery Sciences, Department of Medicine and Division of Pharmacoepidemiology and Pharmacoeconomics, Brigham and Women’s Hospital, Boston, Massachusetts, USA; Center for Healthcare Delivery Sciences, Department of Medicine and Division of Pharmacoepidemiology and Pharmacoeconomics, Brigham and Women’s Hospital, Boston, Massachusetts, USA; Harvard Medical School, Boston, Massachusetts, USA

**Keywords:** Caregiving—formal, Caregiving—informal, Dementia, Digital technology, Medication optimization

## Abstract

**Background and Objectives:**

Care partners are critical for making treatment decisions in persons living with dementia. However, identifying them is challenging, hindering the broader use of interventions, such as those using digital technologies. We aimed to (i) assess the feasibility of identifying and contacting care partners using electronic health record (EHR) systems, and (ii) elicit their perspectives on electronic interventions for deprescribing.

**Research Design and Methods:**

We systematically identified care partners of persons living with dementia ≥65 years of age via structured EHR data in a large health care system. Eligible care partners were contacted by patient portal (if they were an established proxy), mail, and phone to complete a survey.

**Results:**

Of 4,138 eligible persons living with dementia identified, 1,084 (26%) had a care partner name recorded in the EHR. Out of 259 (6%) with sufficient care partner contact information for outreach, 74 (29%) completed the survey. Among care partners, 62 (84%) reported being confident in managing dementia medications, 59 (80%) were willing to stop ≥1 medications, and 43 (58%) were very/extremely interested in using digital tools for decision-making.

**Discussion and Implications:**

Despite the low percentage of care partners with sufficient contact information, reach rates were high for contacted care partners, suggesting feasibility for pragmatic system-level interventions. Most care partners showed great interest in using digital health tools for decision-making and managing medications. Therefore, electronic tools could help with identifying care partners and engaging them. However, scaling up interventions requires better care partner documentation or extracting information from free text.

## Background

Care partners play an important role in the care of people living with Alzheimer’s disease and related dementias (ADRD) and mild cognitive impairment (MCI), including being actively involved in supporting their medication management and making treatment-related decisions (Costa et al., 2015; Lee et al., 2019; Lingler et al., 2016; Noureldin & Plake, 2017; O’Conor et al., 2024). Care partner involvement is particularly important for complex decisions like reducing high-risk medication use for persons living with dementia (Aston et al., 2017; Maidment et al., 2017). Despite the potential benefit and care partners’ willingness to be more involved in medication-related decision making (Sawan et al., 2021), relatively few interventions for persons living with dementia based in outpatient care have explicitly involved caregivers for medication optimization or deprescribing, defined as the stopping or reducing of medications for which risks outweigh benefits (Reeve et al., 2015). Specifically, most prior interventions have focused on other types of caregiver support or general symptom management (Austrom & Lu, 2009; Christie et al., 2019; Green et al., 2020; Pickering et al., 2020; Schneider et al., 2019; Zafeiridi et al., 2018).

The successful engagement of care partners is a crucial component of interventions to deprescribe high-risk medication in persons living with dementia so that any treatment optimization is planned in partnership with patients and their care partners (Farrell et al., 2023; Jansen et al., 2016; Woodward, 2003). However, care partner engagement is challenging for a variety of reasons. First, not all care partners are equally involved in medication management and making deprescribing decisions, and their engagement may also differ depending on care recipients’ cognitive functioning and medication regimen complexities (Gillespie et al., 2014; Huang et al., 2015; Lim & Sharmeen, 2018; Lee, et al., 2019; O’Conor et al., 2021). Often there are multiple care partners and in such situations, persons living with dementia depend on multiple care partners to coordinate care and make treatment decisions (Travis et al., 2000). Second, the existence of care partners, their roles, and contact information are often insufficiently documented in electronic health record (EHR) systems (Dalal & Schnipper, 2016; Ma et al., 2022), which complicates potentially scalable system-wide outreach using structured EHR information. Prior efforts that have attempted to systematically identify care partners in outpatient settings have also often been highly resource intensive (Ma et al., 2022; Van Houtven et al., 2022), which motivated testing a pragmatic identification and electronic outreach approach in outpatient settings that could facilitate the future use of interventions, in particular those using digital technologies.

In addition, although there is evidence that technology-based tools can support care partners in improving their caregiving skills, self-efficacy, and reducing their burden of care (Bradley et al., 2023; Parker et al., 2008; Sohn et al., 2023; Zhai et al., 2023), less is known about their specific perspectives on the acceptability of using technology for managing medications of persons living with dementia and making treatment decisions (Bradley et al., 2023). The framework by Wolff et al. states that care partners are often overlooked in digital health initiatives and emphasizes that we must better identify, engage, and support care partners to facilitate an equitable use of digital technology (Wolff et al., 2024). When designing such tools we must recognize diverse care partner relationships and roles and provide tailored support and resources to care partners (Wolff et al., 2024). Better understanding care partner attitudes toward acceptability of technology for medication management will therefore inform future deprescribing interventions using digital technology aimed at optimizing medication use in persons living with dementia. Because future system-wide electronic deprescribing interventions for persons living with dementia require successful care partner identification and engagement, we aimed to assess the feasibility of identifying and engaging care partners of persons living with dementia via electronic health record (EHR) systems in the outpatient setting and to evaluate reach rates across different strategies with increasing resource intensity (patient portal, mailer, and phone communication). In addition, to inform the nature of a future deprescribing intervention for care partners and persons living with dementia delivered pragmatically through the EHR, we aimed to explore care partners’ perspectives on the acceptability of using technology-based tools (e.g., patient portals, apps, other online support tools) for medication management and deprescribing decisions in their care recipients.

## Method

### Electronic Identification of Care Partners

**Figure 1.**
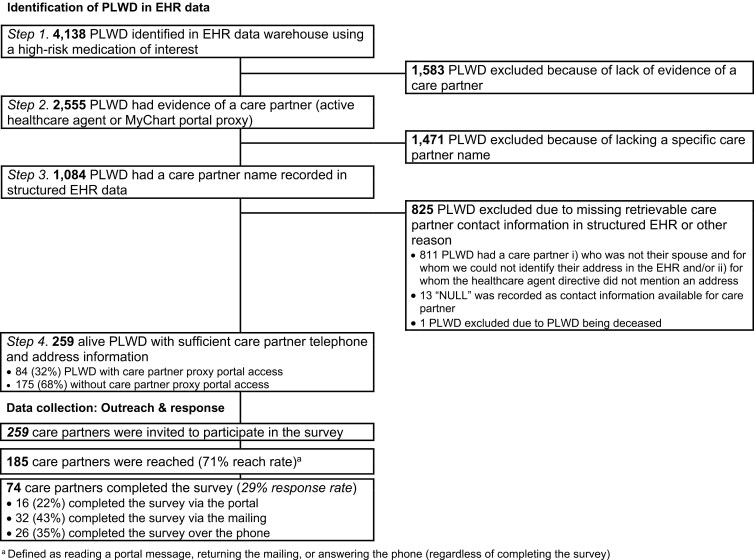
Identification and outreach flow chart.

We used real-time EHR data stored in the Enterprise Data Warehouse (EDW) across 17 Brigham and Women’s Hospital (BWH) and 18 Massachusetts General Hospital (MGH) primary care clinics located in Massachusetts to identify persons living with dementia with a deprescribing opportunity. This was defined as individuals ≥65 years who, in the prior 12 months, had a clinical encounter with a diagnosis codes for ADRD/MCI or a prescription of any ADRD-specific medication in the prior 12 months and had received a prescription for a cognition-affecting medication, specifically benzodiazepines, sedative hypnotic (“Z-drugs”), or anticholinergic medications in the prior 12 months (Step 1) (Figure 1) (Harding et al., 2020; Jaakkimainen et al., 2016). We selected this age group and these medications because they were listed in the American Geriatrics Society Beers Criteria® (“American Geriatrics Society 2019 Updated AGS Beers Criteria® for Potentially Inappropriate Medication Use in Older Adults,” 2019) as medications that should be avoided in older adults (≥65 years) that also can affect cognition.

Among these individuals, we identified who had a care partner listed in structured EHR data, defined as having either (a) a health care agent (HCA) registered and active in the EHR, (b) provided a proxy with access to the MyChart patient portal, or a (c) health care proxy document signed and uploaded as part of the advance care planning documentation (Step 2). Third, among the persons living with dementia identified in Step 2, we confirmed those who had a specific name recorded in structured EHR data for the care partner (Step 3). Lastly, we restricted to persons living with dementia with sufficient care partner contact information (i.e., phone and/or mailing address), so that we could contact these care partners as part of hypothetical clinic outreach intervention (Step 4), which we simulated through the delivery of a brief survey. Any type of care partner-person living with dementia relationship was eligible. If there were multiple care partners with sufficient contact information, we randomly picked one. Of note, email is not an allowable form of initial outreach for research-related projects at BWH and MGH.

We extracted sociodemographic and clinical characteristics of identified persons living with dementia (i.e., age, gender, race, ethnicity, marital status, primary language, cognitive diagnoses, and hospitalization in the 90 days prior to identification) from the EHR data. We also extracted information on the relationship status between care partners and their care recipient (available for those eligible in Step 3) (e.g., wife, husband or spouse, or son/daughter). Lastly, for care partners with proxy portal access, we extracted available information on the care partner’s gender, language, race, ethnicity, age, and educational status.

### Care Partner Survey

We designed a brief survey about care partners’ perceptions about deprescribing as well as their perspectives on the acceptability of the use of technology in the context of medication management and deprescribing. Specifically, the survey contained questions about the sociodemographic characteristics of care partners and persons living with dementia, care partner activities, care partners’ confidence in being involved in the medication management of their care recipient, care partners’ attitudes toward using digital technology to support medication management and deprescribing, and care partner attitudes toward deprescribing medications. Care partner attitudes toward deprescribing were assessed using the care partner questions from the revised Patients’ Attitudes Towards Deprescribing (rPATD) questionnaire, selecting only the questions equivalent to those in the rPATD version for persons living with dementia (rPATDcog), as this selection was most relevant to our population of interest (Reeve et al., 2023; Reeve et al., 2016) (full questionnaire available in Supplementary Appendix 1). “Digital technology” was defined as digital support tools such as the use of patient portals, internet forums, support videos, or devices like personal computers, tablets, and smartphones that are connected to the internet.

### Data Collection and Reach Rates

We delivered this survey in Fall 2023 using a three-phase approach to measure the feasibility of care partner outreach using the identified care partners (Supplementary Figure 1). We designed this phased approach to mimic how a hypothetical deprescribing intervention using digital technology might contact care partners using increasingly resource intensive outreach methods. Specifically, care partners (if they had proxy portal account access) were first contacted via the patient portal with a message sent on behalf of the health system that contained the link to the REDCap survey collection tool. Two weeks after the initial message, we sent one reminder if the survey was not completed. Then, two weeks after the last portal message, care partners with proxy access who had not completed the online survey were mailed a paper version of the questionnaire with a pre-stamped return envelope. Similarly, care partners without proxy access (and therefore were not reachable via the portal directly) were mailed the same paper version of the questionnaire, skipping the portal step. Lastly, three weeks after the letter was mailed, care partners received a phone call if the questionnaire was not completed. Those who completed the survey through any mechanism received a $20 Amazon voucher.

Finally, we measured care partner “reach rates” based on a composite of the following actions, regardless of whether care partners completed the survey: (a) “read rates” of ≥1 portal message, (b) mailing back any part of the mailing (including an empty envelope, excluding any “undeliverable” mailings), and (c) having been reached by phone (answering the phone call and speaking with study staff). These data were collected using REDCap^®^ electronic data capture tools hosted at Mass General Brigham (Harris et al., 2009, 2019).

### Statistical Analysis

We assessed the percentage of persons living with dementia with eligible care partners across the different eligibility criteria. We also used descriptive statistics to analyze the care partner reach rates, survey completion rates, quantitative survey results and sociodemographic characteristics of persons living with dementia, and care partners recorded in the EHR, including those who were reached as well as those who completed the survey. Continuous variables were presented as means and standard deviations (*SD*) and categorical variables as frequencies and percentages. Stata was used to conduct the analyses (version 17) (StataCorp. 2023. Stata Statistical Software: Release 17. College Station).

### Ethical Approval

This research was approved by the Institutional Review Board of the Brigham and Women’s Hospital (2021P002240).

## Results

### Feasibility of Electronic Care Partner Identification and Outreach

Of 4,138 eligible persons living with dementia identified in the EHR, 2,555 persons living with dementia had evidence of a care partner (62%), and 1,084 had a care partner with a specific EHR-recorded name (26%) (overall flowchart shown in Figure 1). Of those, 259 care partners had sufficient recorded contact information and were invited to complete the survey (259 invited care partners/4,138 eligible persons living with dementia = 6%) (Figure 1).

Reach rates were high among persons living with dementia who did have care partners with sufficient contact information. Specifically, 185/259 (71%) of care partners were reached through the composite of portal, mail, or phone. Most care partners with proxy portal access (*n* = 55/84, 65%) viewed the portal message with the survey link itself. Forty one care partners (of 229 who were sent a letter, 18%) mailed any component of the letter back, including nine who mailed the letter back without completing it; only one was returned to the sender as “undeliverable.” Most care partners (who had not previously completed the survey) were reached by telephone (*n* = 123/217, 57%).

In total, 74 of the 259 (29%) invited care partners completed the survey. Of those, 24 were proxy portal users (32%). Sixteen (22%) care partners completed the survey online after receiving the invite via patient portal, 32 (43%) care partners completed the paper questionnaire, and 26 (35%) responded to the survey during a phone call.

### Characteristics of Care Partners and Persons Living With Dementia

Sociodemographic characteristics of persons living with dementia were relatively comparable throughout the identification steps (e.g., gender, age) (Steps 1–3) (Supplementary Table 1). One notable exception was that there was a higher percentage of persons living with dementia with named care partners (Step 3; 14%) who were recently hospitalized than those in the broader population identified in the EHR (Step 1; 7%). Among the care partners invited to participate in the survey (Step 4), the characteristics of persons living with dementia were of similar age compared to those identified in Steps 1–3, but persons living with dementia in Step 4 included more men, White and non-Hispanic individuals, and primary English speakers (Supplementary Table 1). There were also a higher number of persons living with dementia who were married, in a civil union, or with a life partner. The sociodemographic and clinical characteristics of the persons living with dementia whose care partners were invited to participate in the study, reached, and who completed the survey were comparable (Supplementary Table 2). Persons living with dementia of care partner survey respondents were on average 81 years old (*S*D = 7) and 39 (53%) were male. More than 90% were White older adults, and English was the most spoken language.

Of those care partners completing the survey, 46 (62%) were women, 70 (95%) were White, with a mean age of 72 years (*SD* = 11) (Table 1). In addition, 57 (77%) of the care partners who completed the survey were the spouse or life partner of the persons living with dementia. Relationships between care partners and PLDWs were comparable between survey respondents and care partners invited to participate. Forty seven (64%) of care partners reported being the only care partner, 69 (93%) were the health care proxy, most reported having been a care partner for more than two years and almost a third reported spending of 40 hours/week on caregiving activities (Table 2). Among care partners with proxy portal access, the percentage of children of the persons living with dementia was higher, their mean age was lower (65 years, *SD* = 13). Children of persons living with dementia, younger adults, and persons with a higher educational level made up a higher percentage of those filling out the survey via the patient portal. Among the care partners who completed the survey via mailing, the mean age tended to be older and contained more spouses/life partners (Supplementary Table 3).

**Table 1.** Characteristics of Care Partners of People Living With Dementia

Characteristics	Care partners who completed the survey (*n* = 74)	Care partners who were reached (*n* = 185) ^a^	Care partners invited to participate in survey (*n* = 259) ^b^	Care partners with proxy portal access (*n* = 84/259) ^c^
Relationship to care recipient, *n* (%)				
Husband, wife, or partner	57 (77%)	136 (74%)	199 (77%)	28 (33%)
Child	16 (22%)	33 (18%)	39 (15%)	35 (42%)
Friend/family friend	1 (1.4%)	1 (1%)	1 (0.4%)	1 (1%)
Other	0 (0%)	2 (1%)	3 (1%)	3 (4%)
Unknown	0 (0%)	13 (7%)	19 (7%)	17 (20%)
Gender, *n* (%)				
Female	46 (62%)	^d^	^d^	62 (74%)
Male	28 (38%)	^d^	^d^	18 (21%)
Unknown	0 (0%)	^d^	^d^	4 (5%)
Primary language spoken in your home, *n* (%)				
English	72 (97%)	^d^	^d^	73 (87%)
Spanish	1 (1.4%)	^d^	^d^	2 (2%)
Other	1 (1.4%)	^d^	^d^	-
Unknown	0 (0%)	^d^	^d^	9 (11%)
Ethnicity, *n* (%)		^d^	^d^	
Hispanic or Latino/a	2 (2.8%)	^d^	^d^	5 (6%)
Not Hispanic or Latino/a	70 (97%)	^d^	^d^	62 (74%)
Unknown	0 (0%)	^d^	^d^	17 (20%)
Race, *n* (%)		^d^	^d^	
Black/African American	1 (1.4%)	^d^	^d^	1 (1%)
American Indian/Alaskan Native	1 (1.4%)	^d^	^d^	0 (0%)
Asian	0 (0%)	^d^	^d^	1 (1%)
White	70 (95%)	^d^	^d^	60 (75%)
Other	2 (2.7%)	^d^	^d^	6 (8%)
Unavailable	0 (0%)	^d^	^d^	12 (15%)
Age, mean (SD)	72 (11)	^d^	^d^	65 (13)
Education, *n* (%)				
High school graduate or GED completed	5 (7%)	^d^	^d^	12 (15%)
Some college or 2-year degree	17 (23%)	^d^	^d^	5 (6%)
4-year college graduate	18 (24%)	^d^	^d^	32 (40%)
≥4-year college degree	34 (46%)	^d^	^d^	12 (15%)
Other	0 (0%)	^d^	^d^	3 (4%)
Declined	0 (0%)	^d^	^d^	8 (10%)
Unavailable	0 (0%)	^d^	^d^	8 (10%)

^a^Care partners who were considered as “reached” had read minimum 1 portal message, sent the questionnaire back (irrespective of whether they completed the survey) or were reached by phone.

^b^Based on data from electronic health records.

^c^Based on data from electronic health records; data available for 80/84 care partners.

^d^No data available for these variables if the care partners had not completed the survey or completed these fields when becoming a proxy. EHR = electronic health records.

**Table 2.** Care Partner Roles and Activities (*n* = 74)

	*n* (%)
Number of care partners	
Single care partner	47 (64%)
Several care partners	26 (35%)
Missing	1 (1%)
Care partner role	
Primary care partner	56 (77%)
Someone else is the primary care partner	2 (3%)
Shared caregiving responsibilities with someone else or multiple people	14 (19%)
Don’t know	1 (1%)
Missing	1 (1%)
Healthcare proxy for care recipient	
No	4 (5%)
Yes	69 (93%)
Missing	1 (1%)
Decision-making role	
Care recipient is the primary decision-maker	13 (18%)
Care partner is the primary decision-maker	45 (61%)
Share decision-making responsibilities with someone else or multiple people	16 (22%)
Missing	0 (0%)
Hours of caregiving per week	
5 h per week or less	10 (14%)
6–20 h per week	23 (31%)
21–40 h per week	13 (18%)
More than 40 h per week	24 (32%)
Other	1 (1%)
Missing	3 (4%)
How long have you been a care partner for	
6 months or less	1 (1%)
More than 6 months but less than 2 years	11 (15%)
2 years but less than 5 years	30 (41%)
5-10 years	22 (30%)
More than 10 years	9 (12%)
Missing	1 (1%)
Caregiving activities (*multiple responses possible*)	
Personal hygiene and getting dressed	35 (47%)
Eating	29 (39%)
Preparing meals	46 (62%)
Getting in or out of bed or chairs	23 (31%)
Using the toilet, including getting to and from the toilet	18 (24%)
Shopping	50 (68%)
Managing money	53 (72%)
Using telephone	38 (51%)
Provide support so that care recipient can participate in social activities	45 (61%)
Doing heavy work around the house	37 (50%)
Doing light work around the house	44 (59%)
Managing medications	57 (77%)
Making treatment decisions	57 (77%)
Communicating with others	47 (64%)
Translating information	39 (53%)

### Acceptability of Technology Interventions for Care Partners of Persons Living With Dementia for Medication Management and Deprescribing

**Figure 2.**
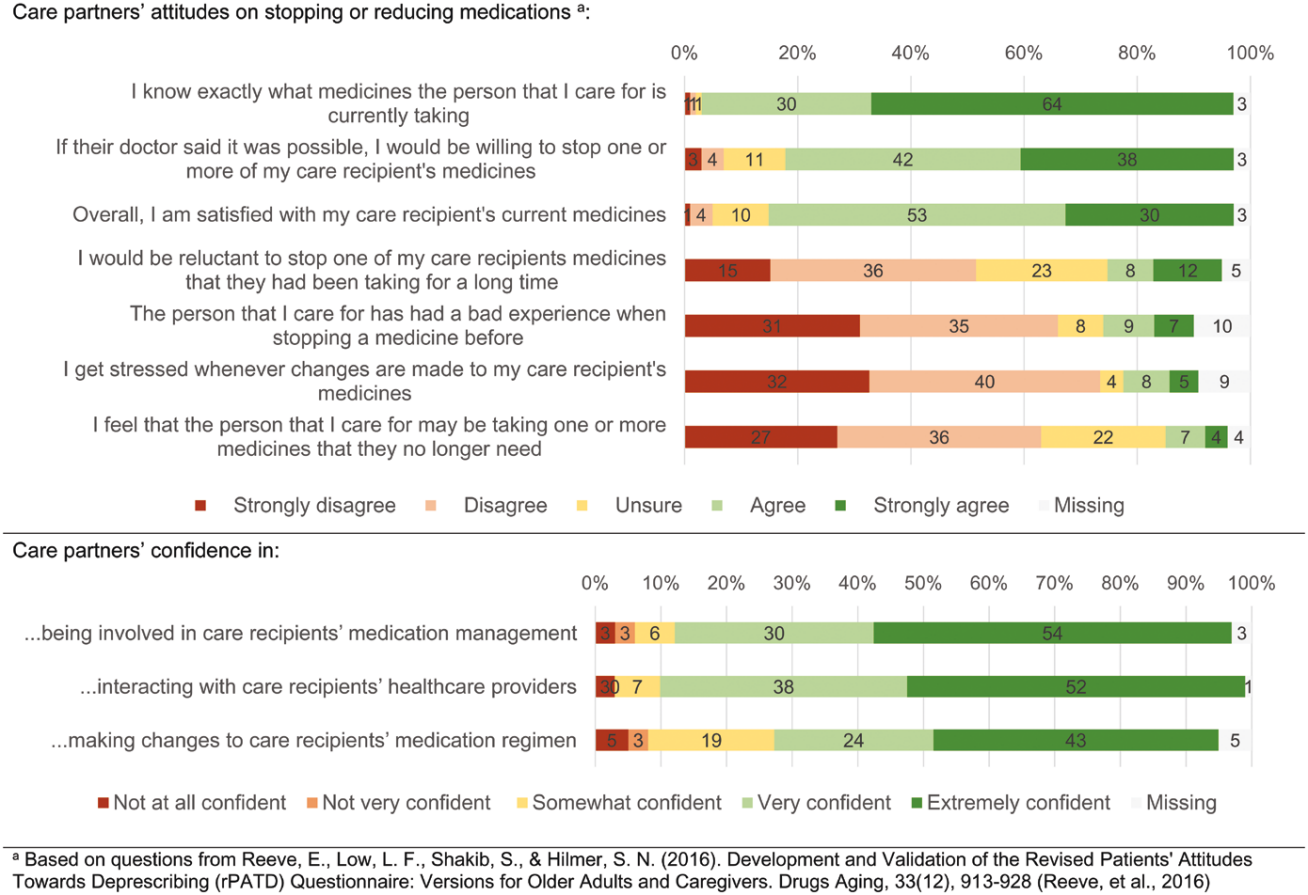
Care partner attitudes towards deprescribing and experiences with optimizing medication use in people living with dementia (*n* = 74).

Most care partners were satisfied with their care recipient’s medication use and reported willingness to make changes to their medication use if recommended (Figure 2). For example, 80% (*n* = 59) of care partners agreed or strongly agreed with the statement: “If their doctor said it was possible, I would be willing to stop one or more of my care recipient’s medicines.” 69 (94%) care partners reported to know exactly what medicines their care recipient was currently taking and only 10 (14%) reported stress whenever changes were made to their care recipient’s medication use. Sixty two (84%) care partners reported being very or extremely confident in being involved in their care recipient’s medication management, and 50 (68%) reported being very or extremely confident making changes to their medications.

For 67 (91%) of the persons living with dementia, care partners reported that a patient portal had been set up. Twenty five (34%) care partners reported that the person living with dementia they care for has access to the portal, 5 (7%) care partners reported that other care partners have access to the portal, and 59 (80%) care partners reported having access to the portal (even if they may not have official proxy access). Care partners reported reasons to explain why they have not set up a portal, which included not being comfortable with technology or not having had time to set up the portal; 77% of care partners reported using the portal for different tasks, with seeing test results (77%), sending messages to healthcare providers before (58%) and after appointments (54%), scheduling appointments (53%), and requesting prescription refills (53%) being the most reported activities (Supplementary Figure 2).

**Figure 3.**
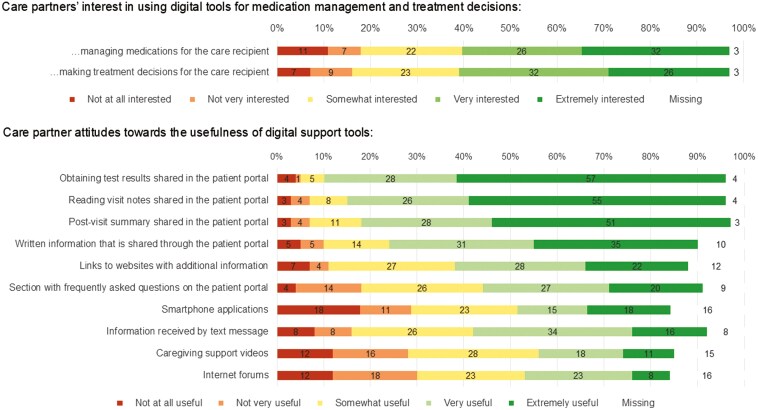
Care partner interest in using digital support tools and perceived helpfulness of different digital support tools (*n* = 74).

Forty three (58%) care partners reported being very or extremely interested in using digital tools for managing the medications of their care recipients, whereas 13 (18%) reported being not at all or not very interested (**Figure 3**). Similarly, 43 (58%) care partners stated being very or extremely interested in using digital tools for making treatment decisions for their care recipient. The digital support tools perceived to be the most useful by care partners were all provided through the patient portal and included obtaining test results, reading visit notes, and post-visit summaries.

## Discussion

Among care partners of persons living with dementia using high-risk medications who responded to our survey, we observed a high confidence in being involved in medication management and making deprescribing decisions and a great interest in using digital health tools for managing medications and making deprescribing decisions, relevant for system-level deprescribing interventions. However, our findings also show that less than 7% of persons living with dementia using high-risk medication in outpatient care had sufficient care partner contact information in structured EHR data to allow for a pragmatic approach to identify and engage care partners via patient portal, mail, and telephone, which is a limiting factor for future care partner engagement efforts and intervention studies. Nevertheless, the reach rates were high for the care partners for whom there was contact information, indicating that an electronic strategy for identification and outreach may be feasible for future deprescribing interventions in this population.

In persons living with dementia, care partner involvement in medication optimization and deprescribing interventions is key, and we found a high care partner confidence in being involved in making medication-related decisions for their care recipients. In line with existing literature, care partners completing our survey showed a high willingness to deprescribe despite being satisfied with their care recipients’ current medication use (Chock et al., 2021; Gadisa et al., 2023; Lee et al., 2022; Rakheja et al., 2022; Roux et al., 2022; Weir et al., 2021). This observation persisted when they were specifically asked about the medication use of persons living with dementia (Reeve et al., 2023). Previous research shows that interventions that engage care partners for medication management and medication optimization increased care partners’ medication knowledge and self-efficacy, but future work should examine the potential impact of these care partner interventions on deprescribing outcomes (Wagle et al., 2018).

Our finding regarding care partner access to patient portals of persons living with dementia is in line with the literature: some have proxy access whereas others report to use the login credentials of the person they care for (Gleason et al., 2023), but do not have official proxy access. This is why they would not be identifiable in an EHR-based approach. Care partners also showed a strong willingness to use digital support tools for medication management and making deprescribing decisions, relevant to deprescribing interventions in persons living with dementia. These findings are plausible considering that previous EHR-based digital health tools to engage care partners, patients, and health care professionals (e.g., in the context of hospital discharge) were found to be feasible and acceptable for participants (Fuller et al., 2020). However, important challenges were identified during their implementation (Frisardi et al., 2022; Fuller et al., 2020), such as care partners’ technological skills, the ideal timing, and a lack of integration with existing systems. These challenges could be due in part to the fact that many prior deprescribing interventions have not involved care partners in the design phase, or even at all in interventions, and may therefore not be tailored to their needs and preferences (Bradley et al., 2023). As such, the willingness of care partners to use technology demonstrated in our survey may translate to an improved ability to involve care partners in future deprescribing interventions.

Beyond their confidence and interest in using tools, our observations of difficulties identifying many care partners for persons living with dementia are somewhat supported by prior literature in other settings. One challenge is that screening for cognitive impairment is underperformed and then subsequent ADRD/MCI diagnoses are under-coded in the EHR (Tan et al., 2023). Successful ways for identifying persons living with dementia include using ICD diagnosis codes for ADRD/MCI in patients’ problem list or past medical history, and/or FDA-approved dementia drugs on medication lists like we did in our care partner identification strategy. Future work on expanding beyond existing diagnoses and treatments is ongoing (Tan et al., 2023; Walling et al., 2023). Another challenge is that enough information on care partners was not readily available structured EHR fields. Others have also observed that information about members of informal care teams is often missing, inaccurate or unreliable (Dalal & Schnipper, 2016). Unfortunately, few healthcare systems have implemented systematic processes for identifying informal care partners due to resource intensity (Ma et al., 2022; Van Houtven et al., 2022). Developers of EHR systems should consistently include these structured fields to allow for systematically assessing care partner information, for example, during annual wellness visits, during hospitalizations or at discharge, or during emergency department visits. For example, de Sola-Smith et al. developed a standardized “Best Possible Caregiver History” approach to assess care partner information during emergency department visits (de Sola-Smith et al., 2024). However, when implementing such an additional documentation of the identity of care partners in the EHR, it must be made sure that they do not result in unreasonably increased time demands and cognitive overload faced by providers (Budd, 2023). Natural language processing could potentially help overcome this by identifying contact information for care partners in unstructured data such as clinic notes and patient messages but has not yet been widely scaled. In addition, systematic processes for identifying informal care partners should be equitable and inclusive (e.g., ensure that structured fields allow for documenting diverse care partners irrespective of their relationship to the patient, make forms available in different languages, etc.). Despite these shortcomings, our findings showed the feasibility of reaching out to the care partners for whom contact information was available in the EHR.

The results from this study could inform the design and implementation of future deprescribing interventions that involve care partners of persons living with dementia in multiple ways. First, our multi-stage approach that escalated in resource intensity, first reaching out to care partners via the portal (if applicable), followed by a letter and a phone call, might be an adaptable blueprint for care partner interventions. Second, we also identified some characteristics of persons living with dementia with contactable care partners that could help tailor future deprescribing interventions. For example, patients who were recently hospitalized more commonly had a care partner recorded with contact information in structured EHR data, suggesting that hospital discharge and the transition to outpatient care may be a potential moment for care partner outreach for an intervention. Lastly a caution for future deprescribing interventions is that persons living with dementia with contactable care partners were more frequently non-Hispanic White and English-speaking compared with those in the broader population. These gaps suggest that more effort should be devoted to identifying the care partners of minority groups and non-English persons living with dementia.

This research also comes with several limitations. First, we were unable to email care partners due to health system restrictions (common across health systems) and were limited to care partners for whom contact information was available in the EHR, which limits the generalizability of our results. Second, our results may overgeneralize toward English-speaking individuals because we only offered the survey in English; ≥95% of respondents were White and non-Hispanic. Unfortunately, we were unable to compare the sociodemographic characteristics of care partners throughout the identification process, as they were not collected in structured EHR systems (unless care partners were an established proxy like the 32% of care partners who responded to the survey). Detailed sociodemographic information is limited to the care partners who participated in the survey (29% of all care partners invited to participate). Third, due to social desirability and volunteer biases, our findings likely overestimate care partners’ willingness to deprescribe and use novel digital tools. The sociodemographic information available is limited to the care partners who participate in the survey (29% of care partners invited to participate). Fourth, the survey response elicited by our outreach strategy may still differ from a deprescribing intervention or other clinical interventions. Finally, our strategy to identify eligible persons living with dementia was based on medication prescribing and not medication use, but is similar to other prior work on EHR data (Walling et al., 2023).

## Conclusion

We found a high care partner interest in deprescribing, confidence to be involved in medication management in persons living with dementia, and interest in using digital health tools for making treatment decisions and managing medication use. Our findings also indicate that our care partner identification and outreach strategy may be a feasible outreach strategy for interventions, such as system-wide electronic EHR-based interventions aiming at deprescribing high-risk medication in persons living with dementia. Among the small percentage of care partners for whom sufficient contact information was available in the EHR, reach rates were high. However, to scale up interventions in this population, strategies to increase the documentation of the identity and engagement of care partners or the ability to extract information from free text would be needed.

## Supplementary Material

gnaf028_suppl_Supplementary_Materials

## Data Availability

Data are available on reasonable request, including the execution of appropriate data use agreements. This study was not pre-registered.
