# Supplementary material for: Feasibility and Acceptability of Engaging Care Partners of Persons Living With Dementia With Electronic Outreach for Deprescribing
Source: Gerontologist. 2025 Jan 28;65(4):gnaf028. doi: 10.1093/geront/gnaf028 (PMC11959457; doi:10.1093/geront/gnaf028)
Supplement: gnaf028_suppl_Supplementary_Materials [file gnaf028_suppl_supplementary_materials.docx]

**Supporting Material**

**Appendix 1. Care partner questionnaire**

This survey is about the involvement of care partners who are involved in making treatment decisions and managing medication use of persons with cognitive impairment, Alzheimer's disease, or any other memory or thinking problems. Please complete the questions below.

Before we get started, we want to share our definition of a caregiver or care partner: *"A caregiver or care partner is someone providing unpaid assistance of care to a family member or friend. Activities requiring assistance may include:*

- *Personal hygiene and getting dressed*
- *Eating*
- *Preparing meals*
- *Getting in or out of bed or chairs*
- *Using the toilet, including getting to and from the toilet*
- *Shopping*
- *Managing money, such as keeping track of expenses or paying bills*
- *Using the telephone, including texting*
- *Providing support so that he/she can participate in social events*
- *Doing heavy work around the house like scrubbing floors, washing windows, and doing yard work*
- *Doing light work around the house like doing dishes, picking up, light cleaning, or taking out trash*
- *Managing medications*
- *Making treatment decisions*
- *Communicating with others*
- *Translating information”*

*Please complete the questions below.*

Eligible respondents who complete the entire survey will receive a $20 Amazon voucher. So that we can follow-up with you and make sure that you'll receive the voucher, please leave your email address (preferred) or phone number at the end of the survey.

**Let us begin with a couple of questions to determine your eligibility to participate in this survey study.**

Do you consider yourself a care partner of at least one family member or friend who has cognitive impairment, Alzheimer's disease, dementia, or any other memory or thinking problems? Check the response that best applies.

□ Yes

□ No *(it seems like you are not eligible to participate, please still send us the questionnaire back using the pre-stamped return envelope)*

*Moving forward, if you care for different persons, please think of the person you spend most hours caring for per week when answering the questions in this survey.*

Which of the following diagnoses/conditions is most applicable to the person you are caring for? Check the response that best applies.

□ Cognitive impairment

□ Alzheimer's disease

□ Dementia

□ Other memory or thinking problems (please specify): __________________________

Does the person you care for receive some of their care at Mass General Brigham (Brigham and Women's Hospital, Massachusetts General Hospital, Brigham and Women's Faulkner Hospital, Newton-Wellesley Hospital, and other Mass General Brigham affiliated community hospitals)?

Check the response that best applies.

□ Yes *(move to the next question)*

□ No *(it seems like you are not eligible to participate, please still send us the questionnaire back using the pre-stamped return envelope)*

Are you minimum 18 years old?

□ Yes *(move to the next question)*

□ No *(it seems like you are not eligible to participate, please still send us the questionnaire back using the pre-stamped return envelope)*

Do you agree to participate in this survey?

The survey is expected to take around 15 minutes.

□ Yes *(move to the next question)*

□ No *(it seems like you are not eligible to participate, please still send us the questionnaire back using the pre-stamped return envelope)*

**⇨ Start of the survey**

How old are you? ___________ (in years)

How do you identify? Check the response that best applies.

□ Man

□ Woman

□ Nonbinary

□ Prefer to self-describe: ____________________________________________

□ Prefer not to say

What race best describes you? Check the response that best applies.

□ Black/African American

□ American Indian/Alaskan Native

□ Asian

□ Native Hawaiian/Other Pacific Islander

□ White

□ Other

What ethnicity best describes you? Check the response that best applies.

□ Hispanic or Latino/a

□ Not Hispanic or Latino/a

What is your relationship to the person you are caring / providing assistance for? I'm their:

Check the response that best applies.

□ Husband, wife, or partner

□ Child

□ In-law (e.g., daughter in-law)

□ Grandchild

□ Brother or sister

□ Other relative

□ Friend / Family friend

□ Other (please specify): ______________________________________

When thinking about the amount of care that this person receives, would you say:

Check the response that best applies.

□ You are the primary care partner

□ Someone else is the primary care partner

□ You share caregiving responsibilities about equally with someone else or multiple people

□ Don't know

How many other care partners are there?

Check the response that best applies.

□ I am the only care partner

□ There are several care partners

➥ How many are there (excluding you)? ___________________

Are you the healthcare proxy for this person?

Check the response that best applies.

□ No

□ Yes

□ Don't know

When thinking about how treatment decisions are made for the person you are caring for, would you say:

Check the response that best applies.

□ The person you are caring for is the primary decision-maker

□ You are the primary decision-maker

□ Someone else is the primary decision-maker

□ You share decision-maker responsibilities about equally with someone else or multiple people

□ Don't know

***In this survey, we are interested in studying the use of digital technology for caregiving activities.***

*"Digital technology" refers to the use of patient portals, internet forums, support videos, or devices like personal computers, tablets, and smartphones that are connected to the internet.*

Has a patient portal (like Patient Gateway) been set up to manage the care of the person you are assisting?

Check the response that best applies.

□ No

□ Yes

□ Don't know

*If* ***no*** *portal is being used*: What is the main reason (or main reasons) why you have not set up your access to the patient portal of the person you are caring for?

□ Not comfortable with technology

□ Don't like doing health communication by computer

□ Haven't gotten around to it

□ The person I care for did not give me access

□ Other (please specify): ________________________________________________

*If a portal* ***is*** *being used*: Who has access to this portal?

□ The person I'm caring for has access to the portal

□ I do have access to the portal

□ Other care partner(s) have access to the portal

□ I do not know who else has access to the portal

*If a portal* ***is*** *being used*: In your experience as a care partner, have you used the patient portal for any of the following? Check all that apply.

□ Schedule an appointment

□ Request reminders about upcoming appointments

□ Conduct a virtual or online visit with a healthcare provider

□ Sent a message to a healthcare provider before an appointment

□ Sent a message to a healthcare provider after an appointment

□ Telephone call request

□ Request a prescription refill

□ See test result

□ Request a referral

□ Other (please specify): ________________________________________________

How do you usually communicate with the healthcare providers of the person that you care for? Check all that apply.

□ Phone

□ Email

□ Patient portal

□ I do not interact with them outside of office visits

□ Other (please specify): _____________________________________________________

In your experience as a care partner, how interested are you in using digital technologies to assist you with:

Check the boxes that apply.

|  | *Not at all interested* | *Not very interested* | *Somewhat interested* | *Very interested* | *Extremely interested* |
| --- | --- | --- | --- | --- | --- |
| Making treatment decisions for the person you are providing care for? |  |  |  |  |  |
| Managing medications for the person you are providing care for? |  |  |  |  |  |

In your experience as a care partner, how useful would you consider the following digital technologies to be for making treatment-related decisions:

Check the boxes that apply.

|  | *Not at all useful* | *Not very useful* | *Somewhat useful* | *Very useful* | *Extremely useful* |
| --- | --- | --- | --- | --- | --- |
| Caregiving support videos |  |  |  |  |  |
| Links to websites with additional information |  |  |  |  |  |
| Information received by text message |  |  |  |  |  |
| Smartphone applications |  |  |  |  |  |
| Internet forums |  |  |  |  |  |
| Written information that is shared through the patient portal |  |  |  |  |  |
| Post-visit summary shared in the patient portal |  |  |  |  |  |
| Reading visit notes shared in the patient portal |  |  |  |  |  |
| Obtaining test results shared in the patient portal |  |  |  |  |  |
| Section with frequently asked questions on the patient portal |  |  |  |  |  |

What could help you improve the communication with the healthcare providers of the person that you are caring for?

***In this section, we would like to ask you a few questions about your previous experiences and your attitudes towards stopping or reducing medications.***

In the last year, can you recall one or several situations in which a healthcare provider (like a doctor) suggested stopping or reducing a medication the person you are caring for was using?

Please choose the response that best applies.

□ Yes

□ No

*Thinking of the situation(s) in which a healthcare provider (like a doctor) suggested stopping or reducing a medication that the person you are caring for was using, how satisfied were you with:*

Check the boxes that apply.

|  | *Very dissatisfied* | *Dissatisfied* | *Neutral* | *Satisfied* | *Very satisfied* |
| --- | --- | --- | --- | --- | --- |
| The oral information provided by the healthcare provider about why this change was needed |  |  |  |  |  |
| The written information material received |  |  |  |  |  |
| Your involvement in the decision-making process |  |  |  |  |  |
| The documentation of the stopping/reduction after the visit |  |  |  |  |  |
| The instructions received for implementing this change after the visit |  |  |  |  |  |

There are no right or wrong answers, please check the box to indicate how strongly you agree with each of the following statements. If there are any questions that you cannot answer, or feel that it doesn't apply to you, please skip it, and move to the next question.

*For the questions that ask about your care recipient's doctor, please think of the doctor that prescribes the most (if not all) of their medicines.*

Check the boxes that apply.

|  | *Strongly disagree* | *Disagree* | *Unsure* | *Agree* | *Strongly agree* |
| --- | --- | --- | --- | --- | --- |
| Overall, I am satisfied with my care recipient's current medicines |  |  |  |  |  |
| I know exactly what medicines the person that I care for is currently taking and/or I have an up-to-date list of their medicines |  |  |  |  |  |
| If their doctor said it was possible, I would be willing to stop one or more of my care recipient's medicines |  |  |  |  |  |
| I feel that the person that I care for may be taking one or more medicines that they no longer need |  |  |  |  |  |
| The person that I care for has had a bad experience when stopping a medicine before |  |  |  |  |  |
| I would be reluctant to stop one of my care recipient's medicines that they had been taking for a long time |  |  |  |  |  |
| I get stressed whenever changes are made to my care recipient's medicines |  |  |  |  |  |

**Before we end, we want to ask you some final questions about yourself and the person you are caring for:**

How old is the person you are caring for? _______ (in years)

What is the gender of the person you care for?

Check the response that best applies.

□ Male

□ Female

□ Nonbinary

□ Other (please specify): _______________

Which of the following activities do you assist, supervise, or remind to this person you are caring for?

Tick all that apply.

□ Personal hygiene and getting dressed

□ Eating

□ Preparing meals

□ Getting in or out of bed or chairs

□ Using the toilet, including getting to and from the toilet

□ Shopping

□ Managing money, such as keeping track of expenses or paying bills

□ Using the telephone, including texting

□ Provide support so that he/she can participate in social events

□ Doing heavy work around the house like scrubbing floors, washing windows, and doing yard work

□ Doing light work around the house like doing dishes, picking up, light cleaning, or taking out trash

□ Managing medications

□ Making treatment decisions

□ Communicating with others

□ Translating information

□ Other (please specify): ________________________________________________

On average, how many hours per day week do you spend providing care or assistance to this person?

Check the response that best applies.

□ 5 hours per week or less

□ 6 to 20 hours per week

□ 21 to 40 hours per week

□ More than 40 hours per week

□ Other (please specify): ________________________________________________

How many medications does the person you are caring for use on a regular basis?

Check the response that best applies.

□ 0

□ 1-5

□ 6-10

□ More than 10

How long have you been assisting or caring for this person? Check the response that best applies.

□ 6 months or less

□ More than 6 months but less than 2 years

□ 2 years but less than 5 years

□ 5-10 years

□ More than 10 years

The following questions refer to your person and your experience as a care partner with managing the health and medicines of the person you are providing assistance to. In your role as a care partner, how confident are you:

Check the boxes that apply.

|  | Not at all confident | Not very confident | Somewhat confident | Very confident | Extremely confident |
| --- | --- | --- | --- | --- | --- |
| .... interacting with his/her healthcare providers |  |  |  |  |  |
| ... being involved in his/her medication management (e.g., obtaining medications, preparing pill boxes) |  |  |  |  |  |
| ... making changes to his/her medication regimen |  |  |  |  |  |

What is the highest grade or year of school you have completed? Check the response that best applies.

□ 8^th^ grade or less

□ Some high school, but did not graduate

□ High school graduate or GED completed

□ Some college or 2-year degree

□ 4-year college graduate

□ More than 4-year college degree

□ Prefer not to say

□ Don't know

What is the most common language spoken in your home? Check the response that best applies.

□ English

□ Spanish

□ Portuguese

□ Chinese

□ Haitian Creole

□ Other (please specify): _______________________

| *Respondents who complete the survey will receive a $20 Amazon voucher. So that we can follow-up with you and make sure that you'll receive the voucher, please leave your email address (preferred) or phone number below.*  *Email address: ________________________________________ (example: john.doe@bwh.com)*  *Phone number: ________________________________________ (example: 617-123-4567)* |
| --- |

| **eFigure 1. Three-phase care partner outreach approach.** |  |
| --- | --- |
| 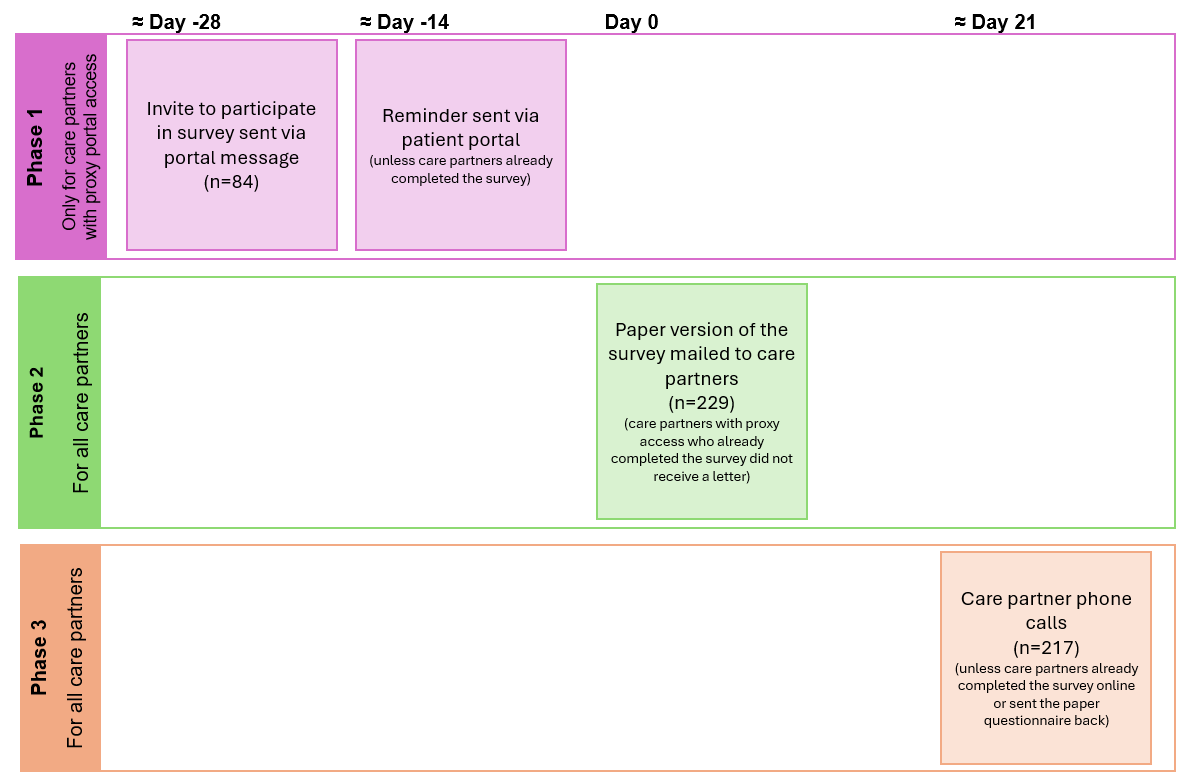 | |

| **eTable 1. Characteristics of people living with dementia throughout the care partner identification process** | | | | |
| --- | --- | --- | --- | --- |
|  | **Step 1.**  Persons living with dementia identified in EHR data warehouse  (n=4,133) ^a^ | **Step 2.**  Persons living with dementia with evidence of a care partner (active healthcare agent or MyChart portal proxy)  (n=2,552) ^a^ | **Step 3.**  Persons living with dementia with care partner name recorded in structured EHR data  (n=1,083) ^a^ | **Step 4.**  Persons living with dementia with complete care partner contact information who were invited to participate in survey  (n=259) |
| **Age, mean (SD)** | 79 (8) | 80 (8) | 81 (8) | 80 (7) |
| **Gender, n (%)** |  |  |  |  |
| Female | 2,733 (66%) | 1,722 (67%) | 735 (68%) | 136 (53%) |
| Male | 1,400 (34%) | 830 (33%) | 348 (32%) | 123 (47%) |
| Unknown | 0 (0%) | 0 (0%) | 0 (0%) | 0 (0%) |
| **Race, n (%)** |  |  |  |  |
| Black/African American | 163 (4%) | 122 (5%) | 53 (5%) | 4 (2%) |
| American Indian/Alaskan Native | 4 (<1%) | 2 (<1%) | 2 (0.2%) | 1 (<1%) |
| Asian | 58 (1%) | 25 (1%) | 18 (2%) | 3 (1%) |
| White | 3,627 (88%) | 2,246 (88%) | 943 (87%) | 236 (91%) |
| Other | 168 (4%) | 100 (4%) | 45 (4%) | 8 (3%) |
| Declined | 48 (1%) | 25 (1%) | 12 (1%) | 2 (1%) |
| Missing | 65 (2%) | 32 (1%) | 10 (1%) | 5 (2%) |
| **Ethnicity, n (%)** |  |  |  |  |
| Hispanic or Latino/a | 264 (6%) | 162 (6%) | 67 (6%) | 8 (3%) |
| Not Hispanic or Latino/a | 3,677 (89%) | 2,309 (90%) | 998 (92%) | 248 (96%) |
| Declined | 25 (1%) | 17 (1%) | 3 (<1%) | 2 (1%) |
| Missing | 167 (4%) | 64 (3%) | 15 (1%) | 1 (1%) |
| **Marital status, n (%)** |  |  |  |  |
| Married/civil union/life partner | 2,191 (53%) | 1,269 (50%) | 503 (46%) | 219 (84%) |
| Divorced/legally separated | 432 (10%) | 284 (11%) | 127 (12%) | 8 (3%) |
| Single | 548 (13%) | 355 (14%) | 170 (16%) | 9 (3%) |
| Widowed | 896 (22%) | 621 (24%) | 274 (25%) | 19 (7%) |
| Declined | 2 (<1%) | 0 (0%) | 0 (0%) | 0 (0%) |
| Missing | 64 (2%) | 23 (1%) | 9 (1%) | 4 (2%) |
| **Language, n (%)** |  |  |  |  |
| English | 3,696 (89%) | 2,294 (90%) | 975 (90%) | 243 (94%) |
| Spanish | 231 (6%) | 143 (6%) | 60 (6%) | 7 (3%) |
| Other | 205 (5%) | 114 (5%) | 48 (4%) | 9 (3%) |
| Missing | 1 (<1%) | 1 (<1%) | 0 (0%) | 0 (0%) |
| **Cognitive diagnoses identified in the EHR data, n (%)** *(Multiple diagnoses per patient were possible)* | | | | |
| Cognitive impairment | 1,778 (43%) | 1,124 (44%) | 468 (57%) | 139 (54%) |
| Alzheimer’s disease | 954 (23%) | 633 (25%) | 276 (26%) | 75 (29%) |
| Dementia | 1,954 (47%) | 1,284 (50%) | 556 (51%) | 128 (49%) |
| Other memory or thinking problems | 1,091 (26%) | 775 (30%) | 377 (35%) | 107 (41%) |
| **Hospitalization in the 90 days prior to the identification date, n (%)** | 284 (7%) | 262 (10%) | 156 (14%) | 26 (10%) |
| This table used data from structured electronic health records (EHR). │^a^ Sociodemographic information unavailable for 5 persons living with dementia of the persons living with dementia identified in the EHR, which is why the n is lower for Steps 1-3 as compared to the study flow chart. | | | | |

| **eTable 2. Characteristics of people living with dementia** | | | |
| --- | --- | --- | --- |
|  | Persons living with dementia of care partners who were invited to participate in the survey (n=259) | Persons living with dementia of care partners who were reached (n=185) ^a^ | Persons living with dementia of care partners who completed the survey (n=74) |
| **Age, mean (SD)** | 80 (7) | 81 (7) | 81 (7) |
| **Gender, n (%)** |  |  |  |
| Female | 136 (53%) | 95 (51%) | 35 (47%) |
| Male | 123 (47%) | 90 (49%) | 39 (53%) |
| Unknown | 0 (0%) | 0 (0%) | 0 (0%) |
| **Race, n (%)** |  |  |  |
| Black/African American | 4 (2%) | 3 (2%) | 2 (3%) |
| American Indian/Alaskan Native | 1 (<1%) | 1 (1%) | 0 (0%) |
| Asian | 3 (1%) | 2 (1%) | 1 (1%) |
| White | 236 (91%) | 169 (91%) | 70 (95%) |
| Other | 8 (3%) | 6 (3%) | 0 (0%) |
| Declined | 2 (1%) | 1 (1%) | 0 (0%) |
| Unknown | 5 (2%) | 3 (2%) | 1 (1%) |
| **Ethnicity, n (%)** |  |  |  |
| Hispanic or Latino/a | 8 (3%) | 5 (3%) | 1 (1%) |
| Not Hispanic or Latino/a | 248 (96%) | 179 (97%) | 72 (97%) |
| Declined | 2 (1%) | 1 (1%) | 1 (1%) |
| Unknown | 1 (1%) | 0 (0%) | 0 (0%) |
| **Marital status, n (%)** |  |  |  |
| Married/civil union/life partner | 219 (84%) | 151 (82%) | 64 (86%) |
| Divorced/legally separated | 8 (3%) | 7 (4%) | 2 (3%) |
| Single | 9 (3%) | 9 (5%) | 2 (3%) |
| Widowed | 19 (7%) | 16 (9%) | 5 (7%) |
| Declined | 0 (0%) | 0 (0%) | 0 (0%) |
| Unknown | 4 (2%) | 2 (1%) | 1 (1%) |
| **Language, n (%)** |  |  |  |
| English | 243 (94%) | 174 (95%) | 72 (97%) |
| Spanish | 7 (3%) | 5 (3%) | 1 (1%) |
| Other | 9 (3%) | 6 (3%) | 1 (1%) |
| **Diagnosis, n (%)** | *Diagnoses identified in the EHR data*  *(multiple diagnoses per patient were possible)* | *Diagnoses identified in the EHR data (multiple diagnoses per patient were possible)* | *Care partners reported the most appropriate diagnosis:* |
| Cognitive impairment | 139 (54%) | 97 (52%) | 21 (28%) |
| Alzheimer’s disease | 75 (29%) | 57 (31%) | 16 (22%) |
| Dementia | 128 (49%) | 101 (55%) | 17 (23%) |
| Other memory or thinking problems | 107 (41%) | 78 (42%) | 20 (27%) |

This table used data from structured electronic health records (EHR). │ ^a^ Care partners who were considered as “reached” had read minimum 1 portal message, sent the questionnaire back (irrespective of whether they completed the survey) or were reached by phone. │EHR = electronic health records.

| **eTable 3. Characteristics of care partners: By form of survey completion (n=74)** | | | |
| --- | --- | --- | --- |
| **Care partner characteristics** | | | |
|  | *Care partners who completed the survey online*  *(n=16)* | *Care partners who completed the paper questionnaire*  *(n=32)* | *Care partners who completed the survey by phone*  *(n=26)* |
| **Relationship to care recipient, n (%)** |  |  |  |
| Husband, wife, or partner | 7 (44%) | 30 (94%) | 20 (76%) |
| Child | 9 (56%) | 2 (6%) | 5 (19%) |
| Friend / Family friend | 0 (0%) | 1 (0.4%) | 1 (4%) |
| Missing | 0 (0%) | 0 (0%) | 0 (0%) |
| **Gender, n (%)** |  |  |  |
| Female | 12 (75%) | 21 (66%) | 13 (50%) |
| Male | 4 (25%) | 11 (34%) | 13 (50%) |
| Missing | 0 (0%) | 0 (0%) | 0 (0%) |
| **Primary language spoken in your home, n (%)** |  |  |  |
| English | 16 (100%) | 32 (100%) | 24 (92%) |
| Spanish | 0 (0%) | 0 (0%) | 1 (4%) |
| Other | 0 (0%) | 0 (0%) | 1 (4%) |
| Missing | 0 (0%) | 0 (0%) | 0 (0%) |
| **Ethnicity, n (%)** |  |  |  |
| Hispanic or Latino/a | 0 (0%) | 1 (3%) | 1 (4%) |
| Not Hispanic or Latino/a | 16 (100%) | 29 (91%) | 25 (96%) |
| Missing | 0 (0%) | 2 (6%) | 0 (0%) |
| **Race, n (%)** |  |  |  |
| Black/African American | 0 (0%) | 1 (3) | 0 (0%) |
| American Indian/Alaskan Native | 0 (0%) | 0 (0%) | 1 (4%) |
| Asian | 0 (0%) | 0 (0%) | 0 (0%) |
| White | 16 (100%) | 30 (94%) | 24 (92%) |
| Other | 0 (0%) | 1 (3%) | 1 (4%) |
| Missing | 0 (0%) | 0 (0%) | 0 (0%) |
| **Age, mean (SD)** | 63 (12) | 76 (8) | 72 (11) |
| **Education, n (%)** |  |  |  |
| High school graduate or GED completed | 0 (0%) | 2 (6%) | 3 (11%) |
| Some college or 2-year degree | 2 (13%) | 9 (28%) | 6 (23%) |
| 4-year college graduate | 3 (19%) | 11 (34%) | 4 (15%) |
| ≥4-year college degree | 11 (69%) | 10 (31%) | 13 (50%) |
| Missing | 0 (0%) | 0 (0%) | 0 (0%) |

| **eFigure 2. Reported patient portal tasks by care partners (n=74)** |
| --- |
|  |

Multiple responses were possible.
